# A Comprehensive Prognostic Model for Colorectal Cancer Liver Metastasis Recurrence After Neoadjuvant Chemotherapy

**DOI:** 10.3389/fonc.2022.855915

**Published:** 2022-06-16

**Authors:** Zhenyuan Zhou, Xin Han, Diandian Sun, Zhiying Liang, Wei Wu, Haixing Ju

**Affiliations:** ^1^ School of The Second Clinical Medical College, Zhejiang Chinese Medical University, Hangzhou, China; ^2^ Department of Hepatobiliary and Pancreatic Surgery, The Second Affiliated Hospital, Zhejiang University School of Medicine, Hangzhou, China; ^3^ Cancer Hospital of University of Chinese Academy of Sciences (Zhejiang Cancer Hospital), Institute of Basic Medicine and Cancer (IBMC), Chinese Academy of Sciences, Hangzhou, China

**Keywords:** colorectal cancer liver metastases, neoadjuvant chemotherapy, histopathological growth patterns, recurrence risk prediction model, nomogram

## Abstract

**Background:**

For patients with colorectal cancer liver metastases (CRLMs), it is important to stratify patients according to the risk of recurrence. This study aimed to validate the predictive value of some clinical, imaging, and pathology biomarkers and develop an operational prognostic model for patients with CRLMs with neoadjuvant chemotherapy (NACT) before the liver resection.

**Methods:**

Patients with CRLMs accompanied with primary lesion and liver metastases lesion resection were enrolled into this study. A nomogram based on independent risk factors was identified by Kaplan–Meier analysis and multivariate Cox proportional hazard analysis. The predictive ability was evaluated by receiver operating characteristic (ROC) curves and decision curve analysis (DCA). Calibration plot were also used to explore the consistency between prediction and reality.

**Results:**

A total of 118 patients were enrolled into the study. Multivariable Cox analysis found that histopathological growth patterns (HGPs) [Hazard Rate (HR) = 2.130], radiology response (stable disease vs. partial response, HR = 2.207; progressive disease vs. partial response, HR = 3.824), lymph node status (HR = 1.442), and age (HR = 0.576) were independent risk factors for disease-free survival (DFS) (*p* < 0.05). Corresponding nomogram was constructed on the basis of the above factors, demonstrating that scores ranging from 5 to 11 presented better prognosis than the scores of 0–4 (median DFS = 14.3 vs. 4.9 months, *p* < 0.0001). The area under ROC curves of the model for 1-, 2-, and 3-year DFS were 0.754, 0.705, and 0.666, respectively, and DCA confirmed that the risk model showed more clinical benefits than clinical risk score. Calibration plot for the probability of DFS at 1 or 3 years verified an optimal agreement between prediction and actual observation. In the course of our research, compared with pure NACT, a higher proportion of desmoplastic HGP (dHGP) was detected in patients treated with NACT plus cetuximab (*p* = 0.030), and the use of cetuximab was an independent factor for decreased replacement HGP (rHGP) and increased dHGP (*p* = 0.049).

**Conclusion:**

Our model is concise, comprehensive, and high efficient, which may contribute to better predicting the prognosis of patients with CRLMs with NACT before the liver resection. In addition, we observed an unbalanced distribution of HGPs as well.

## Introduction

Colorectal cancer (CRC) is the third commonest malignancy, leading to about 0.9 million deaths in 2020 globally (1). The prognosis of colorectal cancer is closely relating to the stage; 5-year survival rate of stage I is more than 90%, whereas that of stage IV with distant metastasis is less than 14% (2). Accumulating evidence has revealed that liver is the most frequent site of CRC metastasis, 15%–25% of patients with CRC having liver metastasis at initial diagnosis and another 18%–25% developing liver metastasis within 5 years of early diagnosis (3, 4). The traditional sandwich approach of neoadjuvant therapy–surgical excision–adjuvant chemotherapy is considered the standard treatment for these patients, but the 5-year survival rate is less than 60% (5–7). Clinical risk score (CRS) is a mature biomarker that was used to predict the risk of recurrence in patients with colorectal cancer liver metastasis (CRLMs) after surgery and direct the preoperative treatment (8). However, CRS focuses on clinical traits of tumor and pays little attention to genetic, pathological, and imaging data of tumor. In addition, CRS assigns one point to all risk factors, without considering the weight of these factors. Furthermore, chemotherapy and follow-up after surgery are an important part of the treatment of CRLMs, but CRS does not seem to focus on this. Therefore, we urgently need a novel, comprehensive, and high-efficient recurrence risk prediction model in clinical practice to guide the postoperative treatment.

For patients receiving neoadjuvant chemotherapy (NACT) before liver resection, the response evaluation criteria in solid tumors (RECIST) are commonly used to estimate the curative effect of NACT and patients’ prognosis, which relies on magnetic resonance imaging (MRI) or computerized tomography (CT) or both (9–11). After hepatectomy, tumor regression grade (TRG), a pathological assessment approach, is utilized to assess the chemotherapy efficacy and outcome (12–14). Recently, histopathological growth patterns (HGPs), new pathology evaluation criteria, have been gradually introduced. Several studies have shown that CRLMs are present in one of three common HGPs referred to as desmoplastic HGP (dHGP), replacement HGP (rHGP), and pushing HGP (pHGP) (15, 16). dHGP presents as the metastatic cancer cells separated with hepatocytes by a margin of fibrous tissue; but in rHGP, cancer cells directly contact with hepatocytes and are in continuity with liver cell; finally, pHGP is the hepatocytes that surround the metastasis, which are pushed away and compressed (15, 17, 18). In terms of prognosis, dHGP is the best, followed by pHGP, and rHGP is the worst (15). Boris et al. further pointed out that patients with non-100% dHGP at liver–tumor interface had significantly lower survival than those with 100% dHGP (19).

Here, we investigated the value of some biomarkers to predict the prognosis of patients with CRLMs accepting NACT before liver resection. Then, we constructed an operational prognostic model for predicting recurrence and stratifying patients. In addition, we compared the purely predictive ability between our model and CRS.

## Materials and Methods

### Patients

Specimens were obtained from patients treated at the Zhejiang Cancer Hospital from May 2009 to December 2019 diagnosed with synchronous or metachronous CRLMs. All lesions presented in the liver before the first chemotherapy. Patients with insufficient basic information, without genetic test results, non-simultaneous radical liver metastases resection, and interventional treatment like radiofrequency ablation or selective hepatic arterial embolization before liver resection were excluded. Patients with extrahepatic metastases before liver resection were excluded from the study. Patients who died within 1 month of hepatectomy were also excluded. The characteristics like age, gender, NACT regimen, targeted drugs, number of metastases, diameter of maximum liver metastasis, time of liver metastasis, invasion depth and lymph node status, RAS and BRAF genes status (RAS gene includes KRAS and NRAS gene), and serum carcinoembryonic antigen (CEA) levels were recorded. Follow-up was done *via* outpatient and telephone visits. Regular liver and lung imaging and serum tumor marker levels were used to monitor tumor recurrence.

### Pathological Assessment of Liver Metastases

All participants gave written informed consent. Liver resection specimens were fixed in formalin, embedded in paraffin, and stained with hematoxylin and eosin (H&E). The slices were independently and blindly evaluated by two experienced pathologists. In case of inconsistent conclusions, the HGPs and TRG results were reviewed by two experts. Positive surgical margins (R1/R2) were defined as residual tumor cells present at or within 1 cm of the resection margins, such patients were excluded. According to the National Comprehensive Cancer Network (NCCN) guideline–recommended evaluation criteria (20–22), TRGs 0 to 2 indicate major response, whereas TRG 3 indicates minor response. HGP analyses were done as the report quoted from the study by Boris et al. (19), with 100% dHGP at tumor–liver interface indicating dHGP and any proportion of rHGP or pHGP at tumor–liver interface indicating non-dHGP.

### Evaluation Criterion of Other Biomarkers

Radiologic evaluation was done on the basis of RECIST version 1.1 (23). We evaluated all measurable lesions and pathological lymph nodes by CT or MRI. A sum of the diameters of longest tumor lesions and short axis for lymph nodes of all target lesions was calculated as baseline sum diameters. The response evaluation of NACT was implemented within 2 weeks after treatment. Next, the sum of shrunken or enlarged diameters of target lesions was determined. Finally, the therapeutic response of the target lesions was obtained by comparing the guideline, including complete response (CR), partial response (PR), stable disease (SD), and progressive disease (PD).

CRS for each patient was determined the report from Fong et al. (8); each of the following criteria assigned 1 point: serum CEA level > 200 ng/ml, diameter of the largest hepatic tumor > 5 cm, number of metastases > 1, and positive lymph nodes and disease-free interval from primary lesion excision to liver metastases < 12 months. The first three clinical data were obtained from the first visit. The total score for each patient was then calculated; scores of 0 to 2 indicated low risk, whereas scores of 3 to 5 indicated high risk.

### Statistical Analysis

Statistical analyses were performed using R and SPSS version 26.0. Ordinal and unordered categorical variables were compared using the Chi-square or Fisher exact test, as appropriate. Survival was evaluated by Kaplan–Meier (K-M) analysis and compared using the log-rank or Breslow test. Disease-free survival (DFS) is defined as the time from liver and primary tumor resection to tumor recurrence. Cox proportional hazard analysis was used to identify independent DFS prognostic factors. Multivariate analysis was completed for factors with *p* ≤ 0.200 after univariate analysis; *p* ≤ 0.05 was statistical significance. The Cox regression consequence was adjusted for confounding bias, checking for multicollinearity, and setting up disordered multi-category variable as dummy variable.

## Results

### Clinical Patient Characteristics

From May 2009 to December 2019, a total of 118 patients with CRLMs who underwent NACT before liver resection were recruited into the study after meeting inclusion criteria. The cohort’s characteristics were summarized on **Table 1**. Most patients were male (66.9%), and 66.9% were < 60 when they first came to the hospital due to colorectal cancer. In addition, 87.3% were in stage T3 or T4, 70.3% have positive lymph nodes, and 63.6% of patients had multiple metastases. Of these, 24.6% had resected lesions with maximum diameters of >5 cm, and 22% were metachronous metastases. Positive serum CEA levels of > 5ng/ml were detected in 72.9% of patients. RAS or BRAF mutations were observed in 50% of patients. With regard to NACT, 69.5% of patients received oxaliplatin-based regimens, 16.1% received irinotecan-based regimens, 17 patients received more than one regimen, and more than half of patients did not receive targeted drugs (61.9%).

**Table 1:** Basic information, tumor characteristics, and pathologic data.

Parameter	No. of patients (n = 118)
Age	
≤60 >60	79 (66.9%) 39 (33.1%)
Gender	
Male Female	79 (66.9%) 39 (33.1%)
Number of metastases	
Single Multiple	43 (36.4%) 75 (63.6%)
Diameter of maximum metastasis	
≤5 cm >5 cm	89 (75.4%) 29 (24.6%)
Time of metastases	
Synchronous Metachronous	92 (78.0%) 26 (22.0%)
Serum CEA	
≤5 ng/ml >5 ng/ml	32 (27.1%) 86 (72.9%)
T stage	
2 3 4	15 (12.7%) 33 (28.0%) 70 (59.3%)
N stage	
0 1 2	35 (29.7%) 51 (43.2%) 32 (27.1%)
NACT regimen	
Oxaliplatin-based Irinotecan-based Multi-regimens	82 (69.5%) 19 (16.1%) 17 (14.4%)
Targeted drug	
Pure NACT Bevacizumab + NACT etuximab + NACT	73 (61.9%) 23 (19.5%) 22 (18.6%)
CRS	
0–2 3–5	55 (46.6%) 63 (53.4%)
Radiology response	
PR SD PD	41 (34.8%) 60 (50.8%) 17 (14.4%)
RAS and BRAF gene status	
Wild-type Mutant	59 (50.0%) 59 (50.0%)
TRG	
0–2 3	44 (28.8%) 84 (71.2%)
HGPs	
dHGP Non-dHGP	19 (16.1%) 99 (83.9%)

Diameter of maximum metastasis: On the first visit, the maximum liver metastasis diameter in contrast-enhanced CT or MRI imaging. Serum CEA, CEA on the first visit; T stage, depth of primary tumor invasion; N stage, lymph node status of primary tumor; Pure NACT, NACT without targeted drugs; CRS, clinical risk score.

According to radiology response, we found that none of patient achieved CR after several cycles of chemotherapy and more than half got SD (50.8%), whereas PD occurred in 14.4% of the patients. Patients with larger tumors tended to have a better regression. Only one (3.4%) patient with > 5-cm lesion progressed, whereas 16 (18.0%) patients with a < 5-cm metastasis progressed after chemotherapy (*p* = 0.001). Moreover, CRS low-risk and high-risk patients were approximately evenly distributed (46.6% vs. 53.4%).

In terms of pathological assessment, 19 (16.1%) patients had a dHGP. Considering the TRG score, patients with TRG 3 accounted for the vast majority (71.2%). Meanwhile, a high correlation between HGPs and TRG was observed, which found that non-dHGP accounted for 95.2% of TRG 3 (*p* < 0.001). Negative lymph node was more common in dHGP (52.6% vs. 25.3%, *p* = 0.017). Moreover, all patients with PD were non-dHGP (*p* = 0.009), and 91% of primary tumor had penetrated the muscularis propria in non-dHGP (*p* = 0.024).

### Disease-Free Survival and Prognostic Factors

The median follow-up was 7.2 months, and the 1-, 2-, and 3-year DFS rates after liver resection were 38.0%, 18.5%, and 13.5%, respectively. A total of 39 (33.1%) patients had not recurrence at the last follow-up.

**Figure 1:**
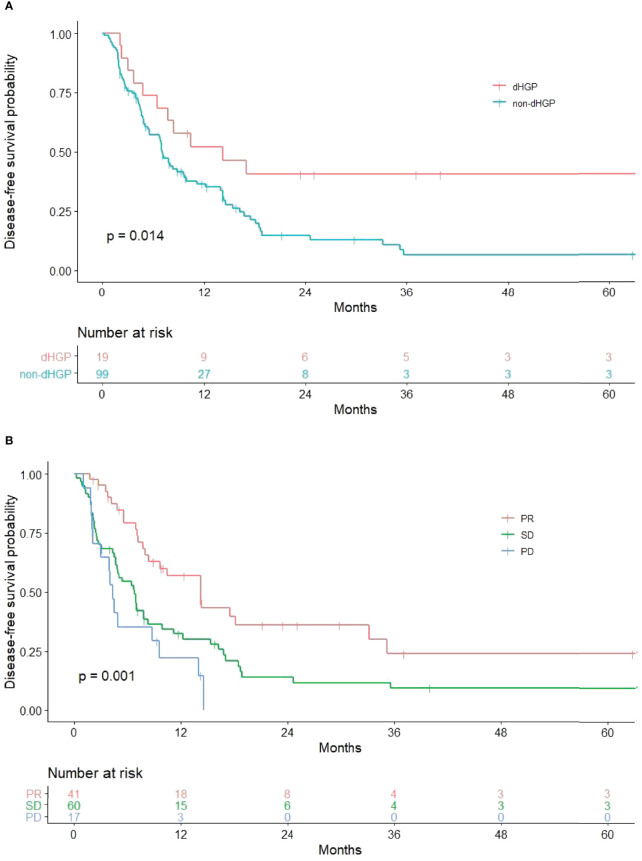
Significant results in univariate analysis. **(A)** Relationship between HGPs and DFS. The non-dHGP is associated with shorter DFS than the dHGP (median DFS = 7.0 vs. 14.2 months, *p* = 0.014). **(B)** Relationship between radiology response and DFS (median DFS for PR vs. SD vs. PD = 14.2 vs. 6.8 vs. 4.3 months, *p* = 0.001).

Results of univariate and multivariate analysis of DFS prognostic factors are shown in **Table 2**. Univariate analysis showed that HGPs (*p* = 0.014, **Figure 1A**) and radiology response (*p* = 0.001, **Figure 1B**) correlated with prognosis. In the multivariate one, HGPs was confirmed as an independent prognostic factor for DFS (HR = 2.130, *p* = 0.048). The median DFS of patients with dHGP was 14 months [interquartile range (IQR): 3–26 months] compared with 7 months (IQR: 6–8 months) in patients with non-dHGP. Additional independent prognostic factors were age (HR = 0.576, *p* = 0.024), lymph node status (HR = 1.442, *p* = 0.015), and radiology response (SD vs. PR, HR = 2.207, *p* = 0.007; PD vs. PR, HR = 3.842, *p* < 0.001). Radiology response was set as a dummy variable in data processing. No multicollinearity was found.

**Table 2:** Univariate and multivariate analysis of predictors of disease-free survival.

Univariate Analysis			Multivariate Analysis	
Parameter	1-year DFS	*p*	*p*	HR (95%CI)
Overall	38.0%			
Age ≤60 >60	35.5% 38.6%	0.158	0.024	0.576 [0.357–0.930]
N stage 0 1 2	51.2% 35.6% 18.3%	0.051	0.015	1.442 [1.074–1.937]
Radiology response SD: PR PD: PR PR SD PD	46.8% 30.2% 14.7%	0.001	0.002 0.007 <0.001	2.207 [1.236–3.942] 3.842 [1.821–8.108]
RAS and BRAF status Wild-type Mutant	38.5% 30.6%	0.089	0.067	1.502 [0.972–2.321]
TRG 0–2 3	40.2% 34.1%	0.085	0.178	0.654 [0.352–1.214]
HGP dHGP Non-dHGP	46.3% 35.0%	0.014	0.048	2.130 [1.007–4.506]

### A New Recurrence Risk Prediction Model

**Figure 2:**
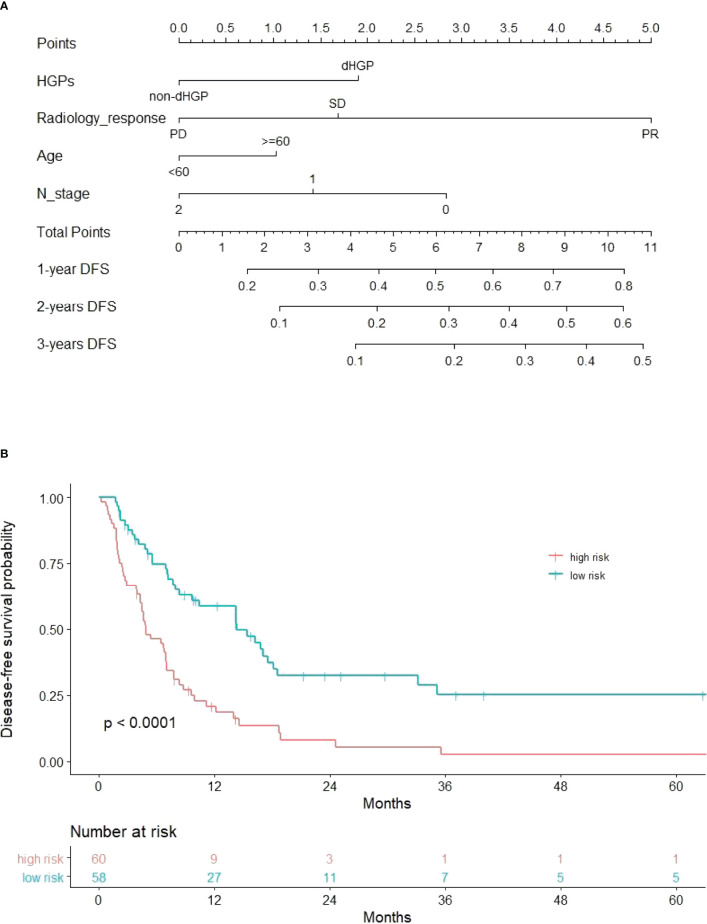
Nomogram and recurrence curve. **(A)** Nomogram incorporating HGPs, radiology response, lymph node status of primary tumor, and age for predicting the DFS of patients with CRLMs. Total points were obtained by summing up individual points from the respective variables, and lower points indicate poorer survival. **(B)** Differences in DFS between high risk and low risk patients (median DFS = 4.9 vs. 14.3 months, *p* < 0.0001).

**Figure 3:**
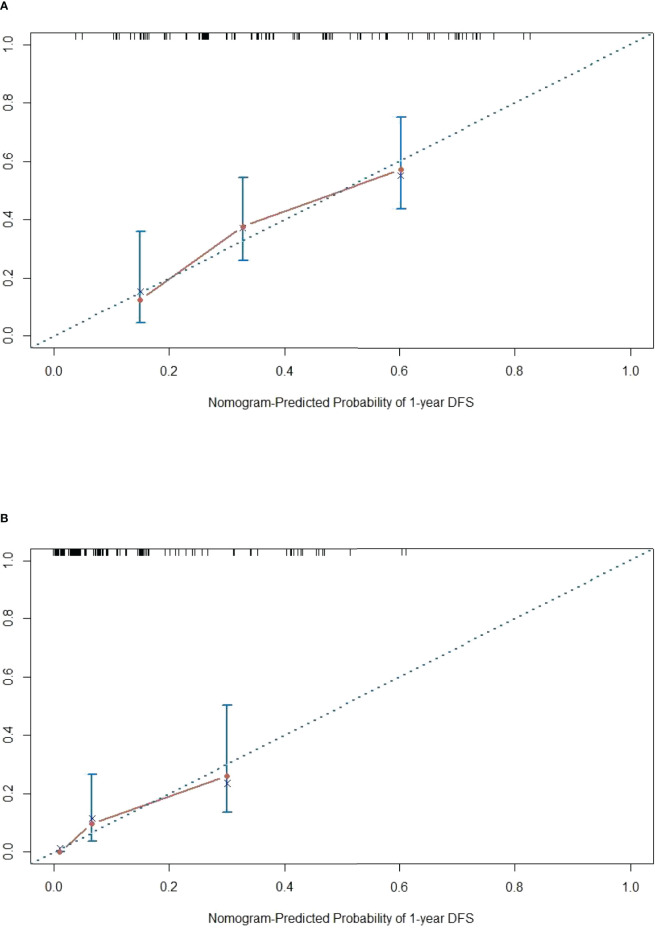
Calibration diagram. **(A, B)** One-year calibration and 3-year calibration diagram for assessment of the nomogram. The nearer distance of red dots to the diagonal line, the more accurate is the prediction of the nomogram.

**Figure 4:**
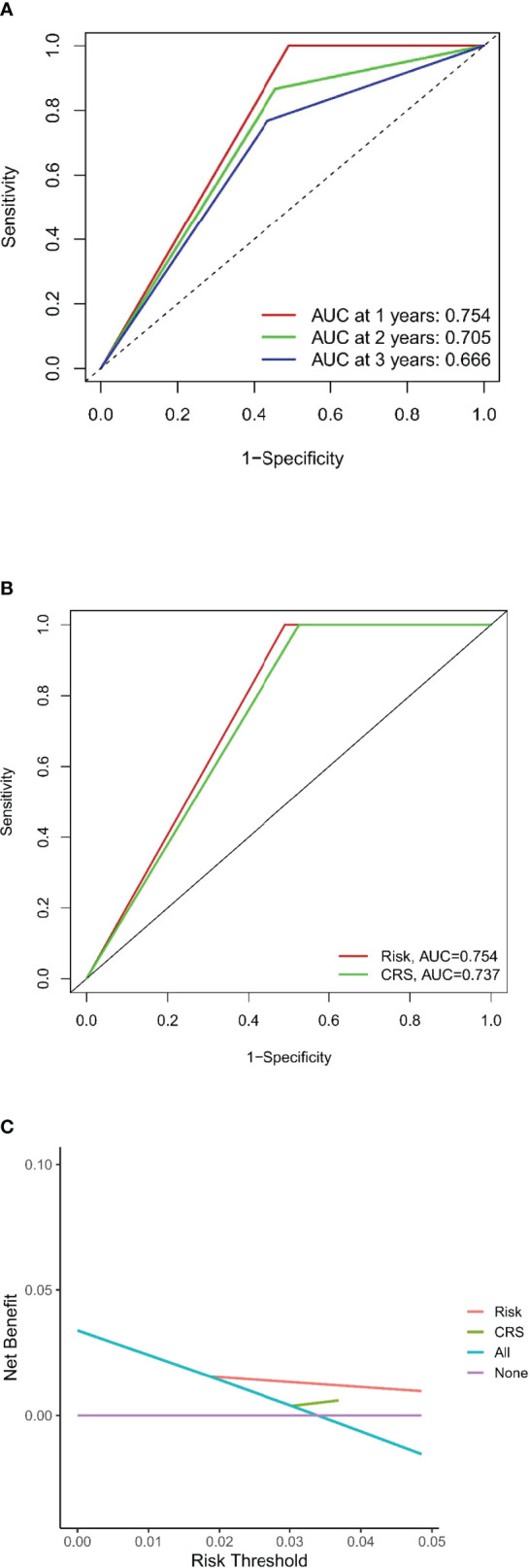
Evaluation of the predictive model and DCA analysis. **(A, B)** ROC curves of the predictive model. **(C)** DCA analysis for new model and CRS.

The DFS nomogram showed in **Figure 2A** based on biomarkers including age, lymph node status, radiology response, and HGPs. Then, we queried patients score from each item and rounded to the nearest whole number before calculating the total score. We then performed a K-M analysis and found that patients’ outcome was improved significantly in score ≥5. Therefore, we classified patients with a score of 5 to 11 as low risk and those with a score of 0 to 4 as high risk. We found DFS to be markedly higher in low-risk relative to high-risk patients (median DFS = 14.3 vs. 4.9 months, *p* < 0.0001, **Figure 2B**). The calibration plot for the probability of 1- or 3-year DFS after surgery demonstrated satisfactory consensus between the prediction *via* nomogram and actual observation (**Figures 3A, B**). Receiver operating characteristic (ROC) curve to predict short- and long-term prognosis based on this risk model revealed area under the curve (AUC) of the risk model 1-, 2-, and 3-year DFS to be 0.754, 0.705, and 0.666, respectively, and the new model has a better AUC than CRS (**Figures 4A, B**). On the basis of **Figure 4C**, the survival decision curve analysis (DCA), which confirmed that the risk model displayed more clinical benefits than CRS.

### Cetuximab Led to a Low rHGP Proportion

In our study, we found a strong correlation between HGPs and cetuximab. rHGP was rare in patients who used cetuximab in NACT before lesion excision. We added a cohort (27 patients) that did not accept NACT before liver resection (control group); thereby, the study cohort was extended to 145 patients. Then, HGP was obtained from every patient in light of the latest international consensus guidelines (15): Taking 50% as the cutoff value, rHGP < 50% rim of liver–tumor interface is non-rHGP and rHGP ≥ 50% rim of liver–tumor interface is rHGP. In univariate analysis, compared with control group, patients who accepted pure NACT without targeted drugs had a lower proportion of rHGP (51.9% vs. 38.4%, *p* = 0.225, **Table 3**). The addition of bevacizumab or cetuximab plus chemotherapy attenuated the rHGP occurrence (51.9% vs. 30.4%, *p* = 0.126; 51.9% vs. 13.6%, *p* = 0.005). Furthermore, cetuximab plus chemotherapy as the one and only independent factor influencing HGPs (HR = 0.173, p = 0.020), no similar effect was observed with bevacizumab (38.4% vs. 30.4%, *p* = 0.491). Transforming patients who treated with pure NACT as control group, cetuximab plus chemotherapy still significantly reduced rHGP incidence (38.4% vs. 13.6%, *p* = 0.030). In addition, cetuximab plus chemotherapy was not an independent factor (HR = 0.267, p =0.055). After ruled out patients who had a more than 50% pHGP or mixture HGP in non-rHGP subgroup, a separate multivariate analysis was calculated. In this further analysis, we also discovered low rHGPs level in cetuximab plus chemotherapy subgroup [HR = 0.176, *p* = 0.025 (vs. non-NACT patients); HR = 0.256, *p* = 0.049 (vs. pure NACT patients)].

**Table 3:** Univariate and multivariate analysis of influence factors of rHGP.

Univariate Analysis			Multivariate Analysis	
Parameter	rHGP	*p*	*p*	OR (95%CI)
CRS 0–2 3–5	31 (42.5%) 21 (29.2%)	0.095	0.490	0.726 [0.293–1.801]
Number of metastases Single Multiple	27 (43.5%) 25 (30.1%)	0.095	0.755	0.863 [0.341–2.181]
Targeted drug Non-NACT Pure NACT Bevacizumab + NACT Cetuximab + NACT	14 (51.9%) 28 (38.4%) 7 (30.4%) 3 (13.6%)	0.042 - 0.225 0.126 0.005	0.121 - 0.322 0.199 0.020	Control 0.629 [0.251–1.579] 0.457 [0.138–1.510] 0.173 [0.040–0.755]
Targeted drug Non-NACT Pure NACT Bevacizumab + NACT Cetuximab + NACT	- - - -	- - - 0.491 0.030	- - - 0.537 0.055	- Control 0.727 [0.264–2.000] 0.267 [0.074–1.028]

## Discussion

The novel recurrence risk prediction model has shown great effectiveness for patients who received NACT before radical primary tumor and liver metastases resection. All the indicators of this model are from the routine preoperative and postoperative examinations of patients, which does not require additional unnecessary medical examinations and will not increase the economic burden of patients. Patient who underwent chemotherapy would evaluate the suitability for surgery using imaging tests before lesions resection, so it is very convenient to acquire every patient’s radiology response. Lymph node status and HGPs assessment are available to perform *via* pathological evaluations on surgical specimens. Compared with other examinations, postoperative pathological examination can more directly reflect the characteristics and the response to preoperative chemotherapy of the tumor and may provide guidance for the selection of postoperative chemotherapy regimens.

CRS system, a basis for the formulation of treatment strategies, provides reliable stratification of patients and is now widely used in clinical practice (24–26). Previous studies have shown no difference in DFS between patients in the low-CRS group who underwent direct surgery and those who received NACT before surgery, but patients in the high-CRS group who received NACT pre-operation had significantly better DFS than those who underwent direct surgery (27). Furthermore, the NCCN guidelines have recommended CRS as the standard stratification for the patients with CRLMs to determine whether patients receive preoperative chemotherapy (20, 21). In clinical practice, there are more patients with high CRS. However, a part of low-CRS patients who have some high-risk factors such as local progression of the primary lesion, the large metastases, or hepatic vascular invasion will also receive NACT. Our model was designed for patients with high CRS and some low CRS who received NACT before surgery. Five criteria of CRS are from the preoperative, focusing on the preoperative personalized treatment of patients with CRLMs. Our model includes both preoperative and postoperative indicators, which can more accurately predict patients’ DFS and provide a reference for clinicians to formulate personalized follow-up and treatment plans for patients after surgery. Nomogram has the inherent advantage of providing different coefficient for different risk factors. Our model and CRS can be used together to demonstrate the precision treatment of cancer, which is in line with the direction of modern cancer treatment. Our model outperforms CRS if it only predicts the prognosis of patients receiving NACT before surgery.

The prognostic value of HGPs was demonstrated again in our study; this is an emerging biomarker and is also the focus of this study. It is also being studied by more and more oncologist and pathologist (28–30). However, the mechanism of different HGPs influencing prognosis is still not clear at present, and it is well known that the angiogenesis of rHGP and dHGP is different. In dHGP, angiogenenic pattern is characterized by a sprouting angiogenesis. As for rHGP, cancer cells replace hepatocytes in the liver cell plates, allowing metastases to integrate in the sinusoidal blood vessels at the tumor–liver interface, without inducing sprouting angiogenesis, which is a process termed “vessel co-option” (15, 17, 18). What is known is that bevacizumab is only effective against sprouting angiogenesis; it may contribute to better outcomes for patients with dHGP (18). Therefore, the underlying molecular mechanisms require further investigation.

Radiology response is the one of the strongest prognostic factors in our study, and the prognosis has a prominent difference between different stratifications. RECIST has been revealed strongly predictive potential in other studies (31, 32). Although TRG did not representing prognostic value in multivariate Cox regression, we detected that almost all (95.2%) TRG 3 were non-dHGP. Compared with dHGP, metastatic tumors with non-dHGP tend to have worse prognostic characteristics in other aspects, such as positive lymph node, tumor progression after chemotherapy, and less tumor regression.

**Figure 5:**
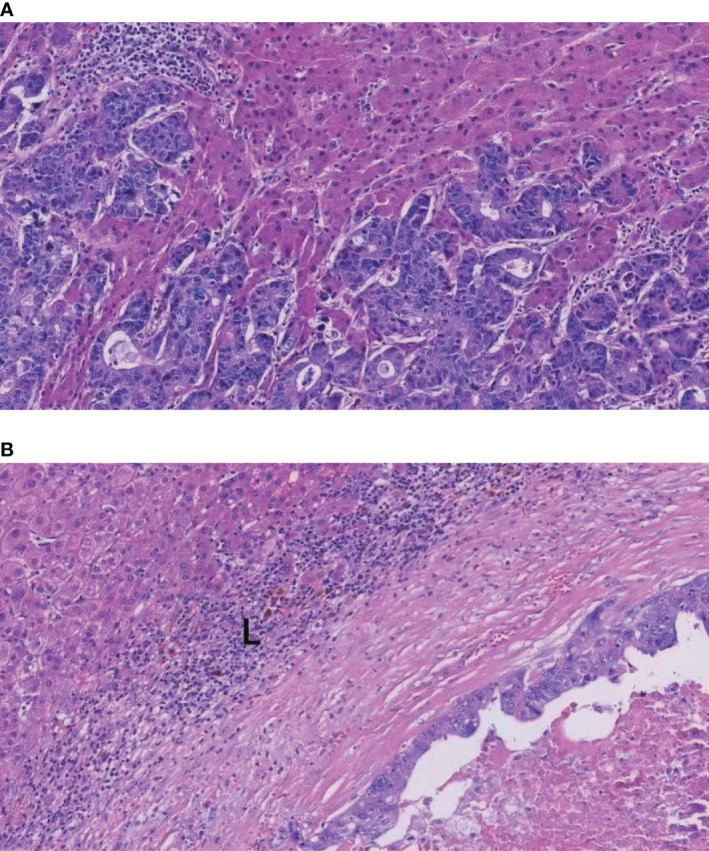
H&E images of the rHGP and dHGP. **(A)** High magnification image of the rHGP. **(B)** High magnification image of the dHGP. L: lymphocyte infiltrate.

Some researchers have pointed out that liver metastases with a more than one growth pattern represented tumors transitioning from one growth pattern to another in some cases (15, 19). Frentzas et al. discovered that chemotherapy plus bevacizumab might increase the proportion of rHGP in patients with recurrence or progression (18, 33). We speculated that cetuximab might convert rHGP to dHGP, resulting in a lower proportion of rHGP in the cetuximab plus chemotherapy subgroup. Tumors with rHGP exhibit reduced immune cells infiltration; in addition, in dHGP, we found dense that lymphocytes infiltration appears in the liver–tumor interface (**Figures 5A, B**). Several studies found that rHGP exhibited reduced CD8^+^ immune cells infiltration (34, 35), and high levels of peri-tumor infiltration by CD4^+^, CD45RO^+^, and CD8^+^ cells would appear in dHGP (15, 34). Höppener et al. reported peritumoral and intratumoral enrichment of cytotoxic CD8^+^ T cells in dHGP as well as a higher CD8^+^/CD4^+^ ratio (36). An immunoglobulin G1 isotype monoclonal antibody (IgG1 mAb), cetuximab, elicits immune reactions like antibody-dependent cell-mediated cytotoxicity (ADCC) involving natural killer cells and T-cell recruitment to the tumor (37). In addition, tumor regression by cetuximab is ADCC-dependent and is mediated by tumor infiltrating CD8^+^ T effector cells. This novel mechanism of ADCC mediated by CD8^+^T effector cells was restricted to IgG1 anti-EGFR mAb (38). Possibly through CD8^+^ T-cell recruitment and improved natural killer cell-mediated ADCC efficiency, cetuximab alters the tumor microenvironment, thereby mediating conversion from rHGP to dHGP.

However, evidence that cetuximab promotes rHGP-to-dHGP conversion is insufficient because we could not prove that the distribution of various HGPs was uniform among the four subgroups before treatment. The best way to demonstrate HGPs conversion is comparing HGPs before and after treatment with cetuximab in the same lesion. There are still many limitations in our study. First of all, this is a retrospective study with weakness related to its study type. Second, although the novel prognostic model has a satisfactory predictive capacity, there is no external validation using data from other experienced centers. Third, because of the low incidence of dHGP, the patients with no dHGP were significantly more than dHGP in our study.

Our model is concise, comprehensive, and high efficient, which may contribute to better predicting the prognosis of patients with CRLMs with NACT before liver resection and provide reference for postoperative treatment of patients. Imaging and pathology data make a fundamental contribution to predicting patient prognosis. We observed an unbalanced distribution of HGPs, and the mechanism of this phenomenon needs further investigation.

## Data Availability Statement

The raw data supporting the conclusions of this article will be made available by the authors, without undue reservation.

## Ethics Statement

The study was reviewed and approved by the ethics committee of the Cancer Hospital of University of Chinese Academy of Sciences (Zhejiang Cancer Hospital), Institute of Basic Medicine and Cancer (IBMC), Chinese Academy of Sciences, Hangzhou, China. The study was performed in accordance with the Declaration of Helsinki. Written informed consent was obtained from all participants for their participation in this study.

## Author Contributions

HJ and ZZ contributed to conception and design of the study. ZZ, WW, and DS organized the database. ZZ and XH performed the statistical analysis. ZZ and XH wrote the first draft of the manuscript. ZL wrote sections of the manuscript. All authors contributed to the article and approved the submitted version.

## Conflict of Interest

The authors declare that the research was conducted in the absence of any commercial or financial relationships that could be construed as a potential conflict of interest.

## Publisher’s Note

All claims expressed in this article are solely those of the authors and do not necessarily represent those of their affiliated organizations, or those of the publisher, the editors and the reviewers. Any product that may be evaluated in this article, or claim that may be made by its manufacturer, is not guaranteed or endorsed by the publisher.
